# Transplantation of Photoreceptor Precursors Isolated via a Cell Surface Biomarker Panel From Embryonic Stem Cell‐Derived Self‐Forming Retina

**DOI:** 10.1002/stem.2051

**Published:** 2015-05-27

**Authors:** Jorn Lakowski, Anai Gonzalez‐Cordero, Emma L. West, Ya‐Ting Han, Emily Welby, Arifa Naeem, Samuel J. I. Blackford, James W. B. Bainbridge, Rachael A. Pearson, Robin R. Ali, Jane C. Sowden

**Affiliations:** ^1^ Stem Cells and Regenerative Medicine SectionUCL Institute of Child HealthUniversity College LondonLondonUnited Kingdom; ^2^ Department of GeneticsUCL Institute of OphthalmologyLondonUnited Kingdom

**Keywords:** Photoreceptor cells, Retina, Surface antigens, Blindness, Retinal dystrophies, Embryonic stem cells, Transplantation

## Abstract

Loss of photoreceptors due to retinal degeneration is a major cause of untreatable blindness. Cell replacement therapy, using pluripotent stem cell‐derived photoreceptor cells, may be a feasible future treatment. Achieving safe and effective cell replacement is critically dependent on the stringent selection and purification of optimal cells for transplantation. Previously, we demonstrated effective transplantation of post‐mitotic photoreceptor precursor cells labelled by fluorescent reporter genes. As genetically labelled cells are not desirable for therapy, here we developed a surface biomarker cell selection strategy for application to complex pluripotent stem cell differentiation cultures. We show that a five cell surface biomarker panel CD73(+)CD24(+)CD133(+)CD47(+)CD15(−) facilitates the isolation of photoreceptor precursors from three‐dimensional self‐forming retina differentiated from mouse embryonic stem cells. Importantly, stem cell‐derived cells isolated using the biomarker panel successfully integrate and mature into new rod photoreceptors in the adult mouse retinae after subretinal transplantation. Conversely, unsorted or negatively selected cells do not give rise to newly integrated rods after transplantation. The biomarker panel also removes detrimental proliferating cells prior to transplantation. Notably, we demonstrate how expression of the biomarker panel is conserved in the human retina and propose that a similar selection strategy will facilitate isolation of human transplantation‐competent cells for therapeutic application. Stem Cells *2015;33:2469—2482*

## Introduction

Many retinopathies, while variable in their etiology, share a common end point, the loss of rod and cone photoreceptors in the retina 1. Inherited retinal degenerations arise from mutations in one of more than 200 different genes 2. In the majority of cases, rod photoreceptors are affected first while loss of cone photoreceptors is secondary due to a trophic dependence on the adjacent rods 3. Worldwide, the number of people blinded by retinal degenerative conditions, including age‐related macular degeneration is estimated to be more than 3.2 million and is predicted to rise as life expectancy increases 4, 5. Unfortunately, the human retina lacks any significant regenerative potential to replace lost photoreceptors; consequently, once these cells have degenerated the resulting visual impairment is permanent. These conditions present a high socioeconomic burden for patients, their families, and the healthcare system 6. While significant progress has been achieved over the past decade in understanding the underlying molecular mechanisms for a range of retinal diseases, current treatment options only delay the onset or decelerate the condition.

To address the current lack of effective treatments, much research effort has been focused on the development of novel therapeutic strategies. Cell replacement therapy, the reintroduction of healthy photoreceptors into the degenerating retina, constitutes such an approach. We and others have previously shown that post‐mitotic, yet immature photoreceptor precursors (PPrs), derived from a defined time window during postnatal development in the mouse can integrate into the existing retinal architecture of the normal and diseased adult retina 7, 8, 9, 10, 11, 12, 13, 14 and contribute to the retinotopic map in the visual cortex 13. Furthermore, we have demonstrated that transplanted rod precursor cells, labelled by the rod‐specific Nrl.GFP transgene, can significantly improve rod‐mediated vision in the *Gnat1^−/−^* mouse model of night blindness 13. The degree of photoreceptor integration appears to be influenced by the host environment as different models of retinal degeneration allow varying levels of cell incorporation 15.

Human embryonic stem cells (ESCs) and induced pluripotent cells (iPSCs) currently represent the most feasible sources of cells for future cell therapies as they are renewable and can in principle give rise to all somatic cell types. While progress has been made in establishing in vitro differentiation protocols for photoreceptor cells, most have not yielded sufficient numbers or the appropriate stage for application in cell‐based therapies 16, 17, 18, 19. Recently, in a landmark study, Sasai and colleagues described an embryoid body‐based three‐dimensional (3D) ESC differentiation system, which recapitulated many aspects of normal retinal development, sparking the prospect of producing sufficient quantities of correctly staged cells for clinical applications 20, 21. Subsequently, we have shown that PPr cells isolated via expression of a Rho.GFP transgene from self‐forming retinae (generated using an adapted Sasai protocol) have the ability to integrate into the healthy and degenerating retinal environment in mice 22. These experiments demonstrated that a stem cell‐based therapy for retinal dystrophies may in fact be possible by combining these new technologies.

One major obstacle preventing translation to the clinic is the lack of strategies to isolate and purify safe and effective cells from complex 3D tissue differentiation systems such as those generated from ESCs or iPSCs. In these cultures, the desired target cells are generated in addition to photoreceptors of inappropriate developmental stages and other undesired retinal and non‐retinal proliferating and nonproliferating cell types. While transplantation‐competent murine donor cells can be isolated relatively effectively from the developing retina via photoreceptor‐specific transgene expression 7, 12, 14, 15, 23, a similar genetic manipulation for clinical application is undesirable given the potential risks of tumorigenicity associated with genetic labelling techniques 24, as well as the need to overcome regulatory hurdles associated with combined cell‐ and gene‐based therapies. The use of conjugated monoclonal antibodies specific to epitopes on the target cells constitutes an alternative to genetic tagging and has already been successfully deployed in clinical applications in the areas of cancer biology and immunology 25, 26, 27. Previously, we identified two cell surface biomarkers, CD73 and CD24, that in combination labelled a (sub)population of postnatal PPr cells and demonstrated that CD73/CD24 positive cells isolated from the postnatal mouse retina integrate efficiently into the normal and diseased mouse eye after subretinal transplantation 28. CD73/CD24 double‐positive rod precursors displayed a significantly higher integration potential than unsorted cells, or rod cells isolated using a conventional Nrl.GFP transgene. However, our data also indicated that additional markers would be necessary for isolation of PPr cells from heterogeneous stem cell differentiation cultures due to the broad distribution of individual cell surface antigens on non‐photoreceptor cells 28. Therefore, here we developed a cell surface biomarker panel of five markers that in combination permits the isolation of post‐mitotic rod precursors from 3D ESC‐derived self‐forming retina. We show for the first time that ESC‐derived rod precursors isolated via a PPr biomarker panel can integrate and mature into the normal or diseased adult mouse retina.

## Materials and Methods

Detailed methods are provided as Supporting Information File 1.

## Results

### Identification of Cell Surface Biomarkers for Photoreceptors

To identify a panel of useful cell surface antigens contributing to the characteristic biomarker signature of transplantation‐competent PPrs, defined as postnatal days 4–8 (P4–P8) 13, we employed a dual approach. First, we examined microarray data of the P4 Nrl.GFP retina 28 for enrichment of genes encoding cluster of differentiation (CD) markers in the Nrl‐expressing rod precursor population compared to other retinal cell types. CD markers represent cell surface molecules useful for cell immunophenotyping and already have widespread clinical application, (e.g., selection of bone marrow stem cells for transplantation 29), due to the availability of well‐established antibodies. Using a twofold cut off to delineate the positive and negative cell populations, we identified 9 and 25 genes for known mouse CD markers that were enriched in rod precursors and other retinal cell types, respectively. An additional 60 CD marker genes were expressed in both populations in the P4 mouse retina (Supporting Information Table 1).

In a second approach, we used flow cytometry to screen postnatal retinal cells from Nrl.GFP mice with a panel of 174 well‐characterized monoclonal antibodies (BD Lyoplate screening panel) to CD markers and identified 15 expressed antigens (>2% in population; Supporting Information Fig. 1A, 1B). A small number of markers labelled subsets of non‐Nrl.GFP cells (e.g., CD309, CD200, CD15, and CD90), while the majority of cell surface antigens were common between retinal cell populations (Supporting Information Fig. 1A, 1B). CD133 and CD73 intensely labelled Nrl.GFP cells compared to non‐Nrl.GFP cells. Comparison of protein expression seen in the lyoplate screen to the respective messenger RNA levels of CD marker genes observed in the microarray analysis (Supporting Information Table 1) showed a widespread congruence, validating gene expression analysis as a useful means of identifying biomarkers for cell selection.

### FACS Analysis of the PPr Biomarker Panel During Retinal Development

We, and others, previously established that CD73 is photoreceptor‐specific in the context of the developing retina, yet it also labels many other cell types, for example, mesenchymal stem cells 9, 28, 30. Furthermore, CD73 is strongly expressed in late‐stage and mature photoreceptors, which have poor transplantation efficiency and would therefore not be useful as a sole selection tool 12, 28. To increase specificity, we examined CD73 colabelling with additional CD markers. Based on their high expression levels in transplantation‐competent Nrl.GFP rod precursors, we selected CD47 and CD133, together with CD73 and CD24, to test as a biomarker signature for positive cell selection (Supporting Information Fig. 1A, 1B; in B, top right hand quadrants of scatter plots show CD marker and Nrl.GFP colabelled cells). All of the CD markers we identified on photoreceptor cells, are known to be expressed on other cell types, but no cell type has previously been defined as expressing this combination of CD markers [CD73(+)CD24(+)CD133(+)CD47(+)] together. To remove potentially harmful mitotically active cells, we utilized the retinal progenitor marker CD15 31, 32, 33 for negative selection, which, as expected, did not show colabelling of Nrl.GFP (Supporting Information Fig. 1A, 1B). Henceforth, the combination of CD73(+), CD24(+), CD133(+), CD47(+), and CD15(−) is referred to as the PPr biomarker panel.

**Figure 1:**
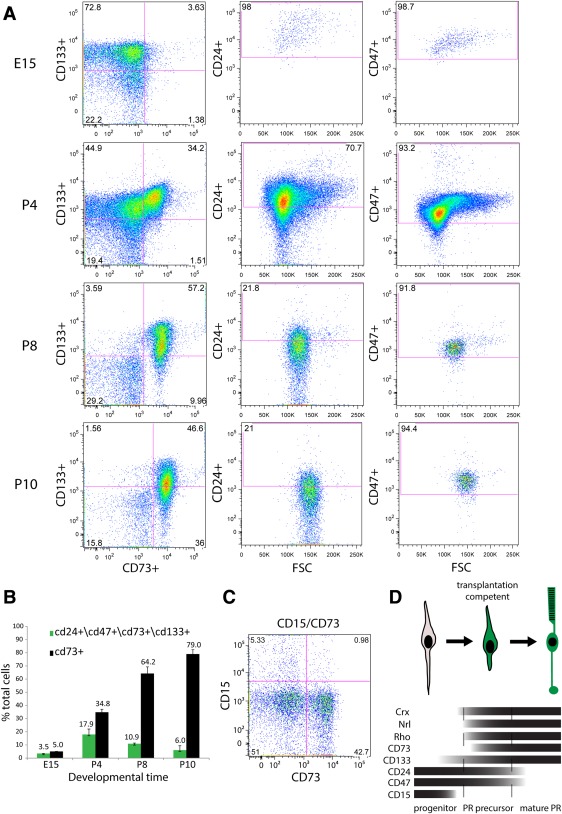
Expression of the photoreceptor biomarker panel during retinal development in the mouse. **(A):** Representative fluorescent‐activated cell sorting (FACS) scatter plots showing coimmunostaining of individual cell surface markers at different time point during retinal histogenesis. Dependent gates are shown from left to right. **(B):** Summary of FACS analysis for combined photoreceptor biomarker panel and CD73 alone. While CD73 alone efficiently labels all post‐mitotic photoreceptor cells, including nonintegration competent adult photoreceptors, the biomarker panel enriches for cells from a narrow postnatal time window, which coincides with transplantation competence. **(C):** Assessment of coexpression of retinal progenitor marker CD15 and CD73 at postnatal day 4. CD15 and CD73 are mutually exclusive during retinal development. **(D):** Schematic showing a comparison of the relative onset of expression of key photoreceptor markers and individual cell surface biomarkers. Vertical bars denote the optimal transplantation competent period for photoreceptor precursors (P4–P8). Abbreviations: FSC, forward scatter; PR, photoreceptor.

The PPr biomarker panel displayed a dynamic expression profile during the course of retinal histogenesis in flow cytometry analyses (Fig. 1A, 1B). At embryonic day 15 (E15), the proportion of cells expressing all four positive selection markers (CD73, CD133, CD24, and CD47) was 3.5% ± 0.1 (Fig. 1B; *n* = 3), due to the low number of CD73 positive photoreceptors in the retina at this point. Over the next few days the proportion of CD73 positive cells increased resulting in an overall colabelling of 17.9% ± 4.1 (Fig. 1B; *n* = 3) for the PPr marker panel at P4, which also represented the peak of coexpression of the biomarkers during retinal development. Among the CD73/CD133 double‐positive cell population, which delineates the developing rods, CD24 and CD47, both markers of immature retinal cells, were also strongly expressed labelling 57.2% ± 17.5 and 96.3% ± 2.3 of the CD73/CD133 double positive population, respectively, at P4 (*n* = 3; see Fig. 1A for representative example). Subsequent developmental stages saw a reduction in the number of PPr panel‐positive cells, with 10.9% ± 0.6 (Fig. 1B; *n* = 3) staining at P8 and only 6% ± 3.3 at P10 (Fig. 1B; *n* = 3). The decrease in colabelling mainly occurred due to the downregulation of CD24 in maturing retinal neurons. While CD47 expression was maintained at P10, it was then rapidly downregulated and absent in mature photoreceptors (Supporting Information Fig. 2A). In a separate set of experiments, colabelling for CD15 and CD73 showed that these two cell populations are mutually exclusive during retinal development indicating that CD15 can be used to remove progenitor cells from cell mixes (Fig. 1C). qRT‐polymerase chain reaction (PCR) and immunohistochemical analysis was also performed on developing retinal samples and showed similar trends of biomarker expression (Supporting Information Fig. 2B, 2C). Taken together our data demonstrate that the PPr marker biomarker panel effectively labels developing but not mature rod photoreceptors (Fig. 1D).

### Characterization of PPr Biomarkers in 3D Mouse ESC Differentiation Cultures

**Figure 2:**
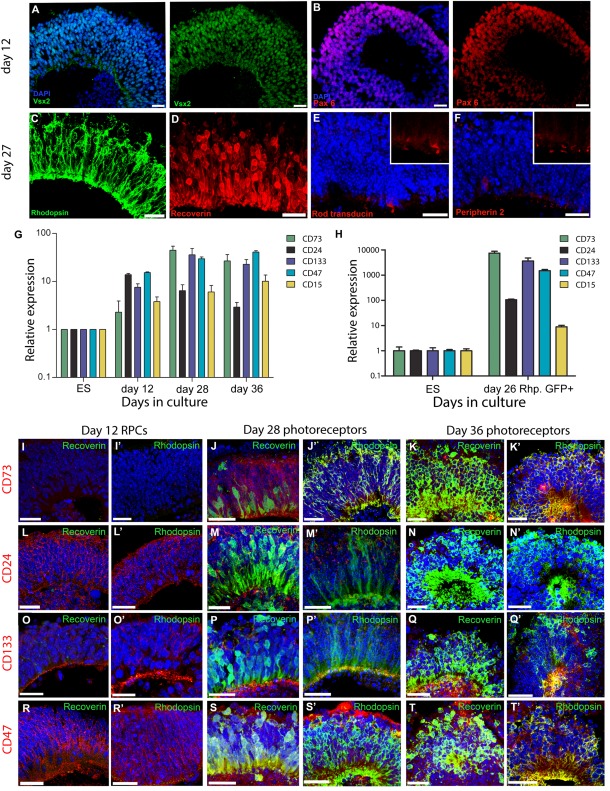
Characterization of photoreceptor precursor (PPr) surface biomarkers on three‐dimensional embryonic stem cell (ESC) retinal cultures. **(A, B):** Day 12 optic vesicle neuroepithelium showing Vsx2 (green, A) and Pax6 (red, B) positive retinal progenitor cells. **(C, D):** Day 28 retinal neuroepithelium regions containing Rhodopsin (green, C) and Recoverin (red, D) positive ESC‐derived photoreceptors. **(E, F)**: Immunohistochemical analysis showing a small number of cells positive for Rod α‐transducin (E) and Peripherin 2 (F) at day 28 of culture. High magnification inserts highlight expression pattern of these markers. **(G):** Quantitative real‐time PCR analysis of ESC retinal cultures demonstrating the expression of PPr biomarker panel over time in culture. **(H):** Expression of the PPr biomarker panel on day 26 fluorescent‐activated cell‐sorted Rhop.GFP+ ESC‐derived rods. **(I):** Immunohistochemical analysis for CD73, CD24, CD133, and CD47 (red), and Recoverin and Rhodopsin (green) on cryosections of ESC‐retinal differentiations at day 12, 28, and 36 of culture. Scale bars: 25 µm. Abbreviation: ES, embryonic stem cell (undifferentiated); PCR, polymerase chain reaction.

PPrs can be generated efficiently using a previously described embryoid body‐based 3D mouse ESC (mESC) differentiation system 20, 22. In this culture system, continuous neuroepithelia are readily produced within 5 days of differentiation and optic vesicle‐like structures appear around days 7–9 (Fig. 2 shows optic vesicle neuroepithelium at day 12). Retinal cell genesis proceeds in a sequence similar to normal retinal development with all neural retinal cell types being present and correctly organized in layers by day 29 of the differentiation procedure, a stage that has been shown to correlate with P4–P8 during mouse retinal development 22.

Consistent with previous observations, we found that at day 12 of differentiation in vitro retinal epithelia showed widespread expression of retinal progenitor markers Pax6 and Vsx2 (Fig. 2A, 2B), suggesting an immature state at this point. By day 27, the majority of cells within the retinal epithelium labelled with the rod markers Rhodopsin and Recoverin and displayed typical photoreceptor morphology (Fig. 2C, 2D). Markers of advanced photoreceptor differentiation, such as rod α‐Transducin and Peripherin 2, were only observed in a few photoreceptors at this point (Fig. 2E, 2F). We assessed the expression profiles of PPr biomarker transcripts in this system using quantitative real‐time (qRT)‐PCR. Expression of all biomarkers could be detected in undifferentiated mESC cultures albeit at relatively low levels. During the retinal differentiation procedure, transcript levels of CD24, CD133, CD47, and CD15 increased from day 0 to day 12, while CD73 transcript levels were significantly increased by day 28; CD24 levels then declined by day 36 (Fig. 2G). We also used an adeno‐associated viral vector (pseudotype 2/9) carrying a GFP reporter under the control of a Rhodopsin promoter (Rhop.GFP) to label rod photoreceptors 22. Importantly, RT‐PCR on FAC‐sorted Rhop.GFP‐positive cells isolated at day 26 of culture confirmed CD73, CD24, CD133, and CD47 expression in the rod population, while negligible levels of CD15 were detected (Fig. 2H).

Next, we determined the spatial distribution of the proteins encoded by CD73, CD133, CD24, and CD47 using immunohistochemistry on tissue sections from early and late ESC differentiation cultures (Fig. 2I–2T). CD73 protein was not detected in early retinal epithelia at day 12 of differentiation but showed intense staining throughout the photoreceptor layer of in vitro retinae at day 28 and day 36, colabelling with rod specific marker Rhodopsin and pan photoreceptor marker Recoverin. CD133, CD24, and CD47 showed a similar wide distribution across the retina during both early (day 12) and later (day 28) stages of differentiation, including the photoreceptor layer and showed overlap with Rhodopsin and Recoverin signals. By day 36, CD24 colabelling with Rhodopsin and Recoverin appeared reduced compared with earlier stages, and compared with the level of colabelling observed for CD73, and CD133 (Fig. 2K', 2N', 2Q'). In addition to the broad cell surface staining also observed with CD24 and CD47, CD133 displayed intense foci of immune‐reactivity at the apical surface of the epithelium (Fig. 2I; white arrowhead), similar to the pattern detected in late postnatal stages 28.

Taken together, these different components of the PPr biomarker panel are present at both transcript and protein levels in retinal differentiation cultures of mESC and exhibit an expression profile broadly consistent with that observed in the developing postnatal mouse retina.

### FACS Profile of PPr Biomarkers During Retinal Differentiation in 3D Cultures

**Figure 3:**
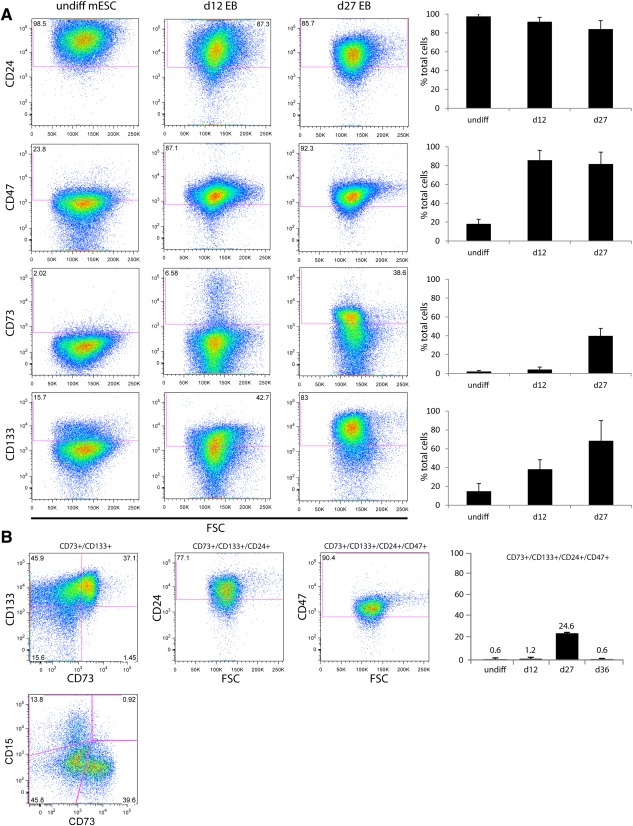
Expression of components of the photoreceptor biomarker panel during mESC differentiation. **(A):** Representative fluorescent‐activated cell sorting (FACS) scatter plots showing the expression of individual cell surface markers in undifferentiated mESC and at day 12 as well as day 27 of the retinal differentiation procedure. A summary of three experiments is shown to the right of the plots. **(B):** FACS analysis of photoreceptor precursor (PPr) biomarker panel at day 12, 27, and 36 of mESC differentiation. At day 27 (scatter plots shown), about 25% of the cells in the differentiating embryoid bodies express the combination of cell surface markers characteristic for postnatal photoreceptors but do not colabel with progenitor marker CD15. By day 36, < 1% of total cell population expresses the PPr biomarker panel. Abbreviations: FSC, forward scatter; mESC, mouse embryonic stem cells.

We next established the percentage of cells labelled by each cell surface antigen at different times of the retinal differentiation protocol, by performing FACS analysis using fluorochrome‐conjugated antibodies (Fig. 3A). In undifferentiated (day 0) mESC cultures CD73 weakly, but consistently, labelled a small number of cells. Labelling then increased significantly to 4.1% ± 2.7 at day 12, and 40% ± 7.9 at day 27 of differentiation consistent with onset of photoreceptor genesis in the in vitro retinae between the two later time points. In contrast, CD24 labelled most if not all cells at day 0 and displayed a slight decrease at subsequent stages of differentiation. CD133 and CD47 displayed a similar staining profile, labelling only a small proportion of cells at the start of the protocol (14.9% ± 8.2 and 18.1% ± 4.9, respectively) and increasing over time to 68.5% ± 21.4 and 81.8% ± 12.5 respectively, at day 27. These data suggest that no single marker would be sufficient to effectively isolate pure and stage‐specific PPrs for the purpose of retinal stem cell therapy.

We next investigated if, in combination, the cell surface biomarkers could be used to isolate cells displaying the typical PPr signature from the differentiation cultures at day 0, 12, and 27. As expected, at day 0 the number of cells expressing the PPr biomarker panel was minute (0.6% ± 0.1 at d0 and 1.2% ± 0.6 at d12, respectively). However, at day 27 of culture 24.6% ± 6.5 of all cells in the in vitro retinae were positive for the PPr marker combination (Fig. 3B). Conversely, the retinal progenitor marker CD15 did not colabel with CD73 (Fig. 3B), indicating that this marker would enhance removal of potential harmful cells prior to transplantation.

**Figure 4:**
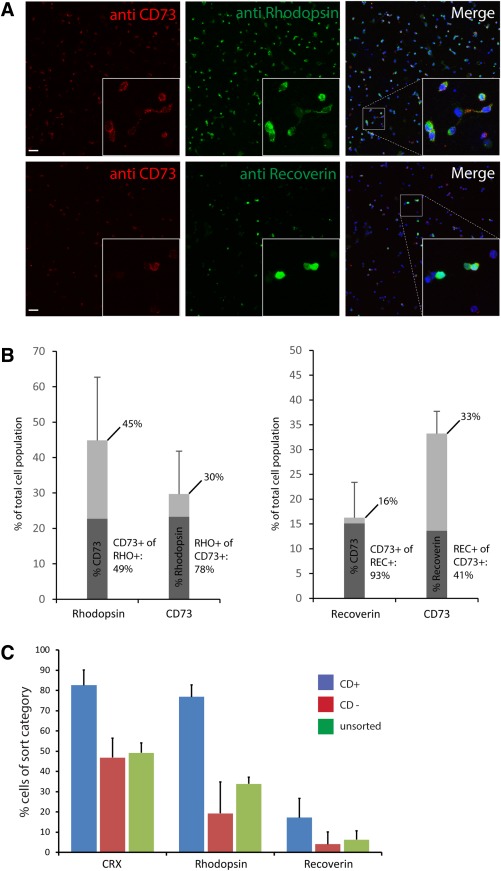
Majority of CD73 positive cells in embryonic stem cell (ESC)‐derived retinal cultures express photoreceptor markers. **(A):** Immunocytochemical analysis of cells from dissociated ESC retinal cultures costained with cell surface biomarker CD73 and photoreceptor markers Rhodopsin or Recoverin. Representative confocal tile scan of plated cells. Inset show high magnification view of indicated area. **(B):** Image analysis software Cellprofiler (Broad Institute, http://www.cellprofiler.org/) was used to determine the number of single or double positive cells from three independent experiments and confirmed by manual counting. Total number of marker positive cells is shown as light bars while darkly shaded bars indicate the percentage of cells costaining for the respective second marker. About 44% of all cells express rhodopsin, 29% CD73, and 16% show Recoverin staining. The majority of CD73 positive cells (∼82%) label with rod photoreceptor marker rhodopsin and ∼40% label with Recoverin. **(C):** Analysis of cells selected from ESC‐derived day 27 retinal cultures using photoreceptor precursor biomarker panel (CD+). Immunocytochemical analysis of CD+ selected cells, CD− and unsorted ESC‐derived cells with photoreceptor markers CRX, Rhodopsin, or Recoverin was used to determine the number of cells expressing each marker (*n* = 3 independent experiments); 82.64 ± 7.53%, 76.83 ± 9.6%, and 17.3 ± 4.9% of CD+ cells labelled with photoreceptor markers CRX, Rhodopsin, and Recoverin, respectively; mean ± SD. Scale bars: 40 µm.

The distribution of CD markers is usually not restricted to one particular tissue or cell type. We, therefore, confirmed the identity of the PPr biomarker panel‐positive cells generated in the 3D retinal culture system using immuno‐cytochemistry (Fig. 4A, 4B). At day 27, retina were dissociated and plated on coverslips to allow investigation of colocalization of CD73 with Rhodopsin or Recoverin on a single cell basis. Rhodopsin and Recoverin were selected as indicators of photoreceptor cell identity with robust available antibodies. We observed that 36.3% ± 14.4 (*n* = 3) of the cells labelled with CD73. As expected, the majority of CD73 positive cells were also strongly colabelled with the rod pigment rhodopsin (78.3% ± 10.5; *n* = 3), confirming the rod photoreceptor identity of PPr biomarker labelled cells generated in the day 27 ESC‐derived retina. Similar analysis conducted using Recoverin showed colabelling of 41% ± 13.6 (*n* = 3) of the CD73 positive cells.

Of the total ESC‐derived population, 45% ± 17.8 (*n* = 3) showed Rhodopsin staining, confirming a robust and reproducible production of rod photoreceptors in this system. Approximately half of the total Rhodopsin‐positive cells, expressed CD73 (49.4% ± 22.1) indicating the presence of PPrs at different stages of development. Of the total ESC‐derived population, 16.2% ± 7.1 (*n* = 3) stained with Recoverin, with the majority of these Recoverin‐expressing cells showing strong CD73 staining (92.9% ± 10.4; *n* = 3).

We also performed immunostaining on cells plated after FACS selection using the PPr biomarker panel (CD+) compared with CD− and unsorted cells from day 27 ESC‐derived cultures. Enrichment for cone rod homeobox gene (CRX), Rhodopsin, and Recoverin‐positive cells was observed in the CD+ population (Fig. 4C). Yields of PPr sorted CD+ cells ranged from 0.41 to 4.89% of ESC cultures at day 27 (*n* = 3) with a viability of 80–90%. Together, these data indicate that the PPr biomarker panel is useful for the isolation of stage‐specific rod photoreceptor cells from ESC‐derived 3D retinal cultures.

### Exclusion of Mitotically Active Cells via PPr Selection

Inclusion of undifferentiated pluripotent stem cells or other proliferating cell populations in cell preparations destined for transplantation presents a serious challenge due to their propensity to result in uncontrolled growth and, in the worst case scenario, elicit the development of tumors 19, 34, 35. It is essential that such cells be removed prior to transplantation to not only eliminate the risk of tumor formation but also increase integration efficiency of genuine photoreceptors as well as to prevent any permanent retinal detachment that could arise from cell masses in the subretinal space 13.

**Figure 5:**
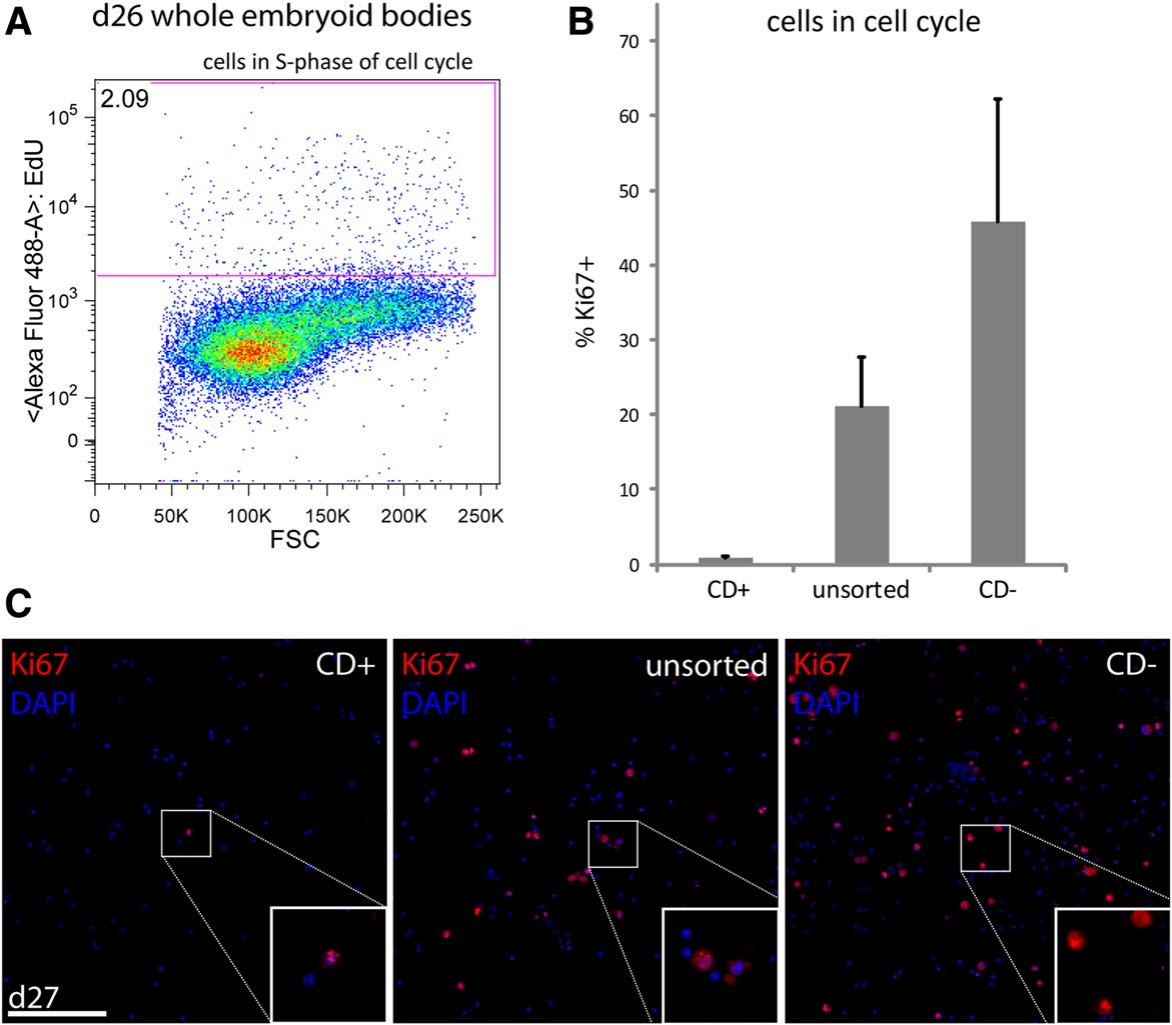
Fluorescent‐activated cell sorting (FACS) via photoreceptor precursor (PPr) biomarker panel eliminates mitotically active cells. **(A):** Representative FACS scatter plot of EdU‐based proliferation assay, following a 2‐hour EdU pulse, showing percentage of d26 embryonic stem cell (ESC) retinal culture cells in S‐phase of cell cycle. Only 2% of cells have incorporated EdU demonstrating that the majority of ESC‐derived cells are post‐mitotic. **(B):** Undifferentiated mouse ESC (15%) were added to dissociated d26 retinal cultures and the resulting cell suspension was sorted via PPr biomarkers. CD(+), CD(−) and unsorted cells were plated for immunocytochemical analysis and the number of Ki67+ cells was determined using Cellprofiler software. **(C):** Summary of Ki67‐based proliferation assay. FACS sorting using PPr biomarkers effectively removed Ki67+, mitotically active cells from the cell sample. DAPI, blue; Ki67, red. Abbreviation: DAPI, 4′6‐diamidino‐2‐phenylindole; FSC, forward scatter. Scale bars: 200 µm.

At day 27, a small number (<2%; *n* = 3) of cells incorporated 5‐ethynyl‐2′‐deoxyuridine (EdU) during S‐phase and 3.25% labelled with Ki67 (*n* = 1) suggesting that this 3D retinal differentiation protocol is generally effective in promoting exit from cell cycle (Fig. 5A). Nevertheless, because of inherent variability within culture preparations, further safeguards to stringently select against any persisting proliferative cells will be required. To test the ability of the PPr biomarker panel to remove mitotically active cells under challenging conditions (i.e., incomplete, less‐efficient differentiation) we added undifferentiated mESC (15%) to dissociated day 26 retinal cultures and determined the number of mitotic cells after FAC‐sorting. No overlap was observed between EdU labelling and PPr biomarker selected cells (data not shown). However, we found that EdU labelling in combination with the five fluorochrome conjugated antibodies to the PPr biomarker panel was not very robust due to limitations in the detection system, therefore Ki67 antibody was used as an alternative quantitative labelling approach for mitotic cells. In these experiments, 21 ± 6.6% (*n* = 3) of unsorted cells from the combined cell population (∼15% undifferentiated ESC: 85% day 27 retinal cultures) showed Ki67 (+) staining (Fig. 5B, 5C). Cells selected from this proliferative population via colabelling with CD73+, CD24+, CD133+, CD47+, CD15− (CD+) contained only a very small number of Ki67(+) dividing cells (0.5% ± 0.23; *n* = 3), demonstrating the effectiveness of the biomarker panel even in the presence of contaminating, undifferentiated cells. On the other hand, a high proportion of proliferating cells were observed in the CD‐ population (46 ± 16.5; Fig. 5B, 5C). These data demonstrate that mitotically active cells are efficiently eliminated from donor cell populations, prior to transplantation, by using the combination of PPr cell surface biomarkers.

### Transplantation of mESC‐Derived Photoreceptors

We next examined the transplantation potential of photoreceptors isolated via the PPr biomarker panel from mESC 3D retinal differentiation cultures. To this end, in a series of experiments, we transplanted 200,000 PPr biomarker FAC‐sorted ESC‐derived rod precursors into the subretinal space of adult wild type, and *Gnat1^−/−^* mice in which rods are nonfunctional due to the absence of the rod α−Transducin protein, pivotal to the phototransduction cascade 36. ESC‐derived rod precursors were sorted at day 27 of 3D retinal differentiation, based on their coexpression of the five specific PPr cell surface biomarkers CD73(+) CD24(+) CD133(+) CD47(+) CD15(−). Two methods were used in order to identify ESC‐derived cells after transplantation. Either wEBs were infected with AAV2/9.CMV.GFP virus several days prior to the experiment, or, alternatively a transgenic mESC line (CBA.YFP ESC; ATCC‐R), with a YFP reporter cassette driven by the ubiquitously active beta‐actin promoter was used.

**Figure 6:**
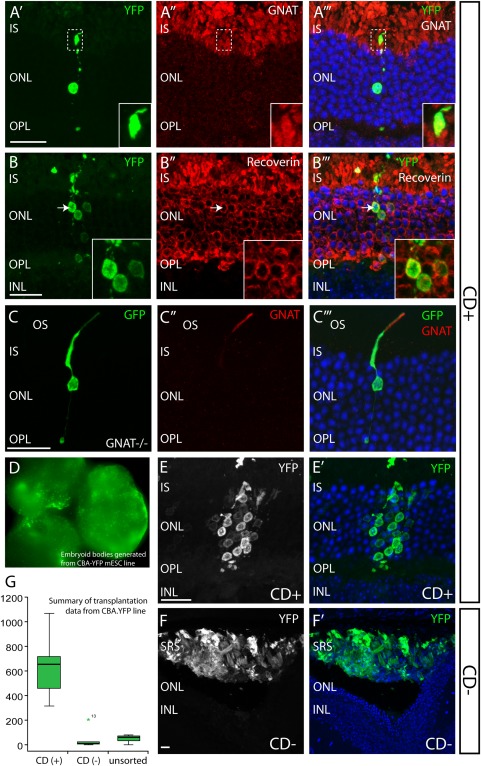
Transplantation of mouse embryonic stem cell (mESC)‐derived, biomarker‐sorted photoreceptor precursors (PPrs) into the adult mouse retina. **(A):** Virally labelled day 27 ESC‐derived PPrs sorted via biomarker panel integrated into *Gnat1^−/−^*adult retina. Anti‐Gnat‐1 immunohistochemistry only labels the outer segments of transplanted cells but not host photoreceptors. **(B):** Whole embryoid bodies from the CBA.YFP mESC line endogenously expressing YFP; day 27 of retinal differentiation cultures were fluorescent‐activated cell‐sorted via PPr biomarker panel and transplanted subretinally into adult wild‐type retinas (B, **C, D, E, F)**. (C, D): Cells expressing the biomarker panel (CD+) integrate into the ONL of the host, while cells from the biomarker negative fraction (CD−) did not integrate and formed large cell clusters in the subretinal space. (E): Summary of data from subretinal transplantation experiments showing numbers of cells integrated within the outer nuclear layer 3 weeks post transplantation. (F): Transplanted cells, which have migrated into the ONL express photoreceptor marker Recoverin. Inset shows a high‐magnification view of the area indicated by arrow. Scale bars: 20 µm. Abbreviations: INL, inner nuclear layer; ONL, outer nuclear layer; OPL, outer plexiform layer; SRS, subretinal space; YFP, yellow fluorescent protein.

Photoreceptors derived from both ESC lines, and selected via PPr biomarker expression (CD+) integrated into the adult mouse retina after subretinal injection (Fig. 6A–6E; Supporting Information Fig. 3). Three weeks post transplantation GFP/YFP labelled cells, with single nuclei and displaying the characteristic rod morphology, were readily visible within the outer nuclear layer (ONL) of recipient mice and were frequently found in small clusters near the injection site (Fig. 6). Rod α−Transducin (Gnat‐1) immunostaining was detected in the outer segments of GFP‐labelled cells integrated into ONL of wild type and in the Gnat‐deficient recipient retina (Fig. 6A, 6C; Supporting Information Fig. 3). In the latter, we observed clear and robust expression of the outer segment protein (Gnat‐1) that was missing in the endogenous rods in the knock out model. The YFP/GFP‐labelled cells displayed typical rod features such as segment formation and spherical synaptic connections in the outer plexiform layer. Furthermore, integrated cells were Recoverin (Fig. 6B) and Rhodopsin positive but did not stain with cone‐specific markers such as RxRγ, sw‐opsin and mw‐opsin (data not shown) demonstrating a rod identity of incorporated cells. Transplantations of cells selected via PPr biomarker expression (CD+) from the mouse postnatal day 8 (P8) Nrl.GFP retina similarly showed integration of GFP+ cells within the recipient wild type ONL three weeks later (Supporting Information Fig. 3). We evaluated the efficiency of transplantation using the PPr biomarker selected ESC‐derived PPrs by counting the number of new GFP‐labelled cells integrated within the recipient ONL. The transgenic mESC line (CBA.YFP), rather than viral labelling, was used for quantification experiments as contaminating viral particles could hypothetically label host photoreceptors 22. Indeed, in experiments using AAV2/9.CMV.GFP virus, we observed a large number of integrated GFP‐labelled cells did not colabel with Gnat‐1, suggesting that either some integrated cells had not yet acquired a mature differentiation state, which can take many days 14, or that a proportion of GFP+ cells were a product of viral labelling of host cells (data not shown).

We found that transplantation of the PPr biomarker positive CBA.YFP ESC‐derived PPrs resulted in integration levels significantly higher than unsorted cells (Fig. 6G; median for CD**+** = 654 cells, range of total number of integrated cells per retina = 315–1,068, *N* = 7), and similar to those previously reported with virally labelled Rhop.GFP ESC‐derived rods 22. By contrast, transplantation of biomarker negative cells (CD−), or unsorted day 27 ESC cultures demonstrated only poor integration abilities (Fig. 6F; median for CD− = 9 cells, range 0–204, *N* = 6; median for unsorted day 27 cultures cells = 60, range 0–80, *N* = 6). We observed integration levels for P8 PPr biomarker positive cells similar to those for ESC‐derived PPr biomarker positive cells (median = 210 cells, range 22–3,039, *N* = 9). In all ESC transplants, non‐integrated GFP+ ESC‐derived cells typically persisted in the subretinal space 3 weeks after injection (Supporting Information Fig. 3C shows cells in subretinal space in low magnification view of Fig. 6E). In transplants of biomarker negative cells (CD−), we frequently observed large subretinal cell masses that contained unidentified cell types of diverse morphologies (Fig. 6F) but little or no integration. Immmunostaining for Pax6 and GFAP, markers of immature neurons and glial cells, respectively, and Ki67 for mitotically active cells, did not label significant numbers of cells in the PPr biomarker panel negative subretinal cell masses at 3 weeks post transplantation (data not shown). Taken together, these data demonstrate that FAC‐sorted ESC‐derived rod precursors selected via the PPr biomarker panel from dissociated synthetic retinae can integrate effectively, and significantly more efficiently than the unsorted ESC‐derived cells or PPr biomarker negative populations.

### Conservation of Biomarkers in the Developing and Mature Human Retina

**Figure 7:**
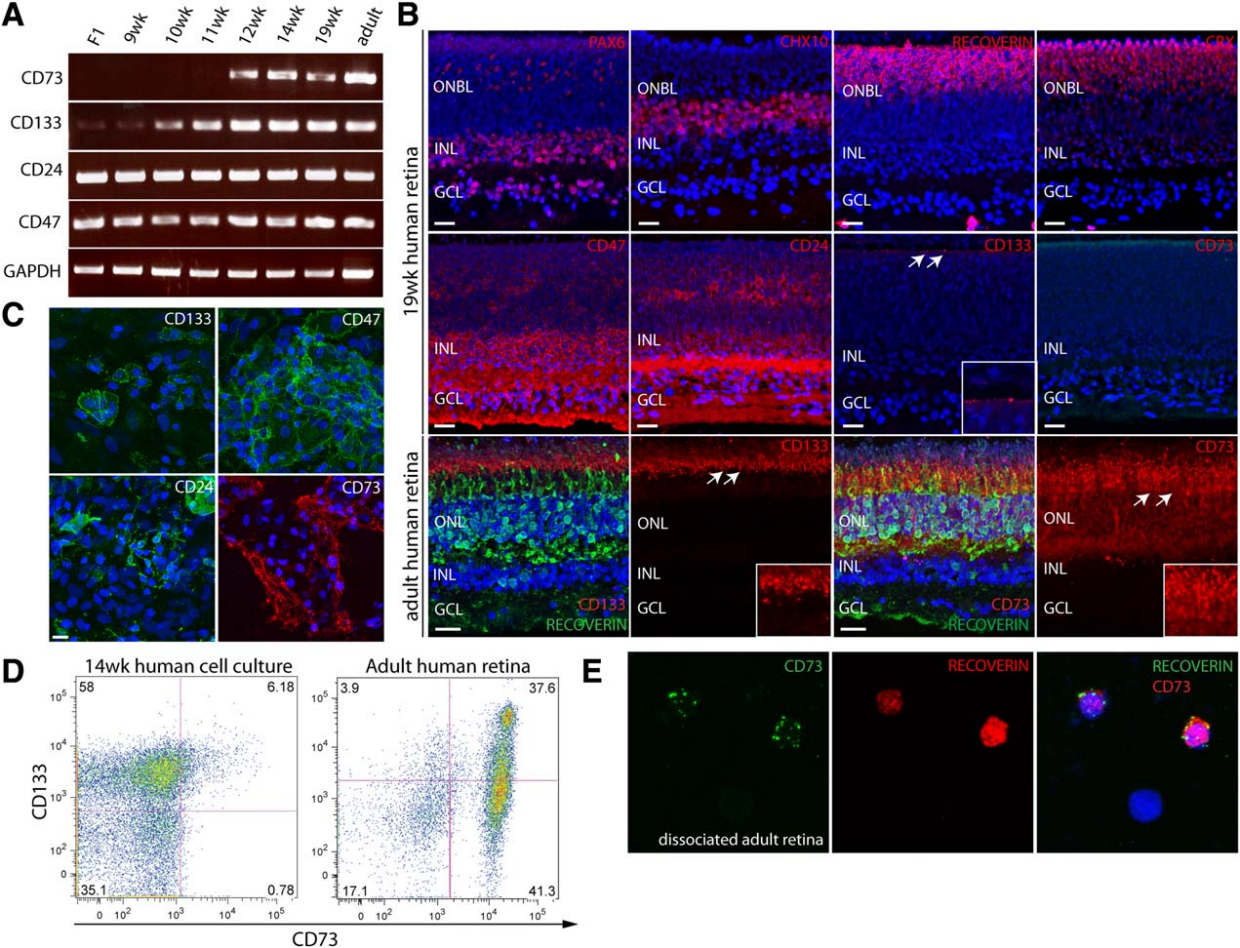
Expression of photoreceptor precursor biomarker panel components in the human retina. **(A):** Real‐time PCR analysis showing the expression of biomarkers at different stages of development in the human retina. CD24 and CD47 transcripts are strongly expressed prior to the F1 stage, while CD133 shows only weak levels of expression and CD73 is absent. CD73 transcripts are first observed at 11 weeks of gestation correlating with the onset of photoreceptor development. **(B):** Immunohistochemistry analysis of cell surface and key retinal markers at 19 weeks of gestation. CD24 and CD47 display a widespread expression pattern, labelling cells in all retinal layers. CD133 immunostaining is restricted to punctate labelling at the apical surface of the developing outer nuclear layer, whereas CD73 staining is absent at this stage. White arrows indicate region of higher magnification inset for CD133 and CD73. **(C):** Immunocytochemical analysis of 14‐week cultured fetal human cells. Cells expressing CD133, CD47, and CD24 are abundant at the beginning of culture, CD73 positive cells only begin to appear after 2 weeks in culture. **(D):** Fluorescent‐activated cell sorting analysis of cultured fetal cells at 14 weeks and adult human retinas. A small number of CD73/CD133 double positive cells are present in primary cell cultures derived from 14‐week retinas. In the adult human retina, ∼78% of cells express CD73 and ∼40% colabel with CD133, consistent with the murine retina. **(E):** Colabelling immunocytochemistry of dissociated adult retinal cells showing all CD73+ cells express the photoreceptor marker Recoverin. Abbreviations: GAPDH, glyceraldehyde‐3‐phosphate dehydrogenase; GCL, ganglion cell layer; INL, inner nuclear layer; ONBL, outer neuroblastic layer; ONL, outer nuclear layer. Scale bars: 20 µm.

To test the usefulness of the biomarker panel for clinical application we investigated the expression of CD73, CD133, CD24, and CD47 in the human retina. RT‐PCR revealed CD24 and CD47 were abundantly expressed even at the early stages of retinal development (8 weeks gestation) and, in contrast to the murine retina, remained at similar levels in the mature tissue (Fig. 7A). CD133 was expressed at low levels during weeks 9, 10, and 11 but increased thereafter and was strongly expressed at the adult stage. In contrast, CD73 mRNA was not detected until 12 weeks of gestation and was present at all subsequent stages of development and in the mature retina.

In order to establish the spatial distribution of the biomarker proteins, we performed immunohistochemistry on cryo‐sections prepared from human fetal and adult retinal tissue. At 10, 13, and 19 weeks of gestation, CD24 and CD47 proteins were detected in all retinal layers (Fig. 7B; Supporting Information Fig. 4) including the developing outer nuclear layer, which contained CRX and Recoverin positive PPr cells. While CD73 transcript was present from 12 weeks onward, no immunostaining was observed during the fetal stages, suggesting a post‐transcriptional control mechanism. In contrast, CD133 protein was visible starting from 10 weeks in a punctate pattern at the apical surface of the retina, abutting the interface with the retinal pigmented epithelium. qRT‐PCR analysis of expression of the PPr biomarker panel genes in human retinal samples showed expression of CD47 and CD24 during fetal stages, but decreased expression in the mature retina (Supporting Information Fig. 4B), in line with the reduced labelling of mature photoreceptors by these markers in the human and mouse retina (Supporting Information Figs. 2 and 4A).

While CD73 protein appeared to be absent in the native fetal human retina, a small number of CD73 immuno‐positive cells were observed in differentiation cultures derived from 14‐week‐old primary retinal cells (Fig. 7C). Approximately 6% of cells in these primary cultures colabelled with CD73 and CD133 in FACS analysis, whereas 39.4 ± 4.76% of cells in the adult human retina were positive for this dual marker combination (Fig. 7D). The total number of cells in the adult retina labelling with CD73 was 74 ± 4.7%, consistent with the total number of photoreceptors (Fig. 7D). Furthermore, in immunocytochemical analysis of dissociated retinal cells all CD73‐positive cells in the adult human retina coexpressed Recoverin, indicating a photoreceptor identity (Fig. 7E).

Taken together, our findings demonstrate that the expression patterns and relative onset of biomarker expression with respect to retinal differentiation is similar between mouse and human. The lack of available human fetal tissue beyond 19 weeks of gestation prevented us from pinpointing the exact onset of CD73 protein expression in the human retina; most rod photoreceptors are generated after 19 weeks. However, our observations are consistent with findings in the murine retina, which showed onset of CD73 protein expression in the postnatal time period when the majority of the rod photoreceptors are born and then sustained expression in photoreceptors.

## Discussion

Cell replacement therapy for retinal disease is a very promising therapeutic strategy currently under investigation, the goal of which is the transplantation of stem cell‐derived cells into the diseased retina, either to substitute photoreceptor cells lost through the disease process and replace disease genes, or to delay or prevent the loss of the remaining cells 37, 38. We and others have demonstrated that PPrs can be introduced into the normal and degenerating rodent retina via subretinal injection and that transplanted rods make appropriate synaptic connections to the remaining inner retinal cells 7, 8, 9, 10, 15. Furthermore, correctly integrated cells have been shown to confer low light visual function in a mouse model of retinal degeneration and signals generated by these cells were projected to visual processing areas in the brain 13.

The most promising sources of donor cells for a future therapeutic application are hESCs and iPSCs; however, the successful translation of this approach to the clinic is critically dependent on the development of methods for the isolation and purification of optimal stage precursor cells. The use of mixed cell populations carries the risk of tumorigenesis due to the presence of mitotically active stem cells, and even inappropriately staged photoreceptors reduce integration efficiency and, therefore, would result in a suboptimal clinical outcome. It is, therefore, critical that a cell selection strategy should enable the specific isolation of wild type cells, which are committed to the photoreceptor lineage, but which have not yet fully matured, as well as excluding proliferating cells.

In order to meet the need for stringent cell selection and avoiding genetic manipulation of cells, we have developed a panel of five useful photoreceptor biomarkers that can be effectively utilized to isolate transplantation‐competent rod precursors from 3D retinal differentiation cultures of mESC. In this study, we utilized existing CD markers for which fluorochrome‐conjugated antibodies already exist for application in FACS protocols. Although we have not formally proved that all five selected CD markers are necessary and sufficient, this study demonstrates for the first time the successful application of a CD marker signature for isolation of PPrs from differentiated ESCs. In future work, it may be possible to develop antibodies suitable for FACs for additional markers identified in photoreceptors by microarray analysis 28, 39. We selected CD73 and CD133 to confer photoreceptor specificity in the context of ESC‐derived retinal differentiation cultures and CD24 and CD47 to enrich for cells equivalent to young, postnatal cells. In addition, CD15 (SSEA‐1) was included for negative cell selection. We showed that the biomarkers for positive cell selection (CD73, CD133, CD24, and CD47) have a peak of coexpression that correlates with the window of transplantation competence (P4–P8) for donor cells isolated from the developing retina. Importantly, the expression of individual biomarkers in mESC differentiation cultures followed the same pattern seen in the developing retina, with 25% of cells in day 27 cultures displaying the biomarker signature of transplantation‐competent rod precursors. The majority of CD73‐expressing cells (∼85%) in day 27 cultures colabelled with the rod marker Rhodopsin. Our data indicate that the PPr biomarker selection panel strongly enriches for immature photoreceptors in the context of this very heterogeneous ESC‐derived retinal cell culture system.

Consistent with this conclusion, we showed that subretinal transplantation of cells selected via the PPr biomarker panel (CD+) resulted in significantly higher integration levels compared to unsorted cells or cells that did not label with the positive‐selection markers (CD−). While integrated CD+ cells displayed the typical rod morphology and labelled with Recoverin, CD− cells rarely integrated and instead formed substantial cell masses with varying morphology. These observations illustrate heterogeneity present in the ESC retinal culture system and the effects of inclusion of non‐photoreceptor cells in cell preparations. Significantly, we showed that the PPr biomarker panel selection excludes proliferating cells, as assessed by Ki67 labelling, even in samples comprising more than 10% mitotically active stem cells.

This study brings together for the first time the use of photoreceptor cell sorting strategies using CD markers, and new ESC‐derived self‐forming retinal cultures to isolate transplantation‐competent cells without genetic modification. This is an important milestone toward the development of clinical photoreceptor cell therapy. The biomarker‐sorted cells maintained viability and showed integration levels similar to those observed using genetically labelled ESC‐derived populations 22. As the integration levels achieved with the ESC‐derived cells and the PPr biomarker selection panel were lower than previously reported experiments using CD73/CD24 selected cells (median: 10,899; range: 544–32,826 28; mean: 2,199 ± 1,006 cells per retina 9,) and Nrl.GFP selected cells (mean: 16,759 ± 1,705 cells per retina 13), isolated from the developing retina, further optimization and fine tuning of differentiation and isolation protocols will be required to maximize the transplantation outcome. Variability in transplant outcomes as reflected in the range of integrated cell numbers, possibly due to variation in host inflammatory responses and surgical delivery of cells 14, 40, 41 also needs to be resolved in future studies. Based on our previous demonstration of restoration of visual function in the *Gnat1* ^−/−^ model containing ∼25,000 newly integrated cells from the developing retina 13, we estimate that a 40‐fold increase in the number of integrating ESC‐derived cells will be required to demonstrate robust restoration of rod function in a mouse model in vivo.

Lastly, the fact that the expression of components of the photoreceptor biomarker panel was conserved in the developing and adult human retina suggests that our cell selection approach may be applicable for isolation of cells for clinical transplantation from human ESC/iPSC retinal culture systems. Taken together, we have identified and tested, for the first time, a set of five cell surface biomarkers that are useful for the enrichment of transplantation‐competent rod photoreceptor cells from pluripotent stem cell‐derived self‐forming retina. These findings define an approach that we anticipate will be broadly applicable for the isolation of photoreceptor cells for clinical therapy.

## Author Contributions

J.L.: conception and design, collection and/or assembly of data, data analysis and interpretation, manuscript writing, financial support; A.G.‐C.: conception and design, collection and/or assembly of data, data analysis and interpretation, manuscript writing; E.W.: conception and design, collection and/or assembly of data, data analysis and interpretation; Y.‐T.H.: collection and/or assembly of data, manuscript writing; E.W.: collection and/or assembly of data; A.N.: collection and/or assembly of data; S.J.I.B.: collection and/or assembly of data; J.W.B.B.: collection and/or assembly of data; R.A.P.: conception and design, collection and/or assembly of data, manuscript writing, financial support; R.R.A.: conception and design, manuscript writing, financial support; J.C.S.: conception and design, data analysis and interpretation, manuscript writing, final approval of manuscript, financial support.

## Disclosure of Potential Conflicts of Interest

The authors declare no potential conflicts of interest.

## Supporting information

Supplementary Information

Supplementary Information

Supplementary Information

Supplementary Information

Supplementary Information

Supplementary Information
