# Supplementary figures and images for: Transplantation of Photoreceptor Precursors Isolated via a Cell Surface Biomarker Panel From Embryonic Stem Cell‐Derived Self‐Forming Retina
Source: Stem Cells. 2015 May 27;33(8):2469–82. doi: 10.1002/stem.2051 (PMC4862023; doi:10.1002/stem.2051)

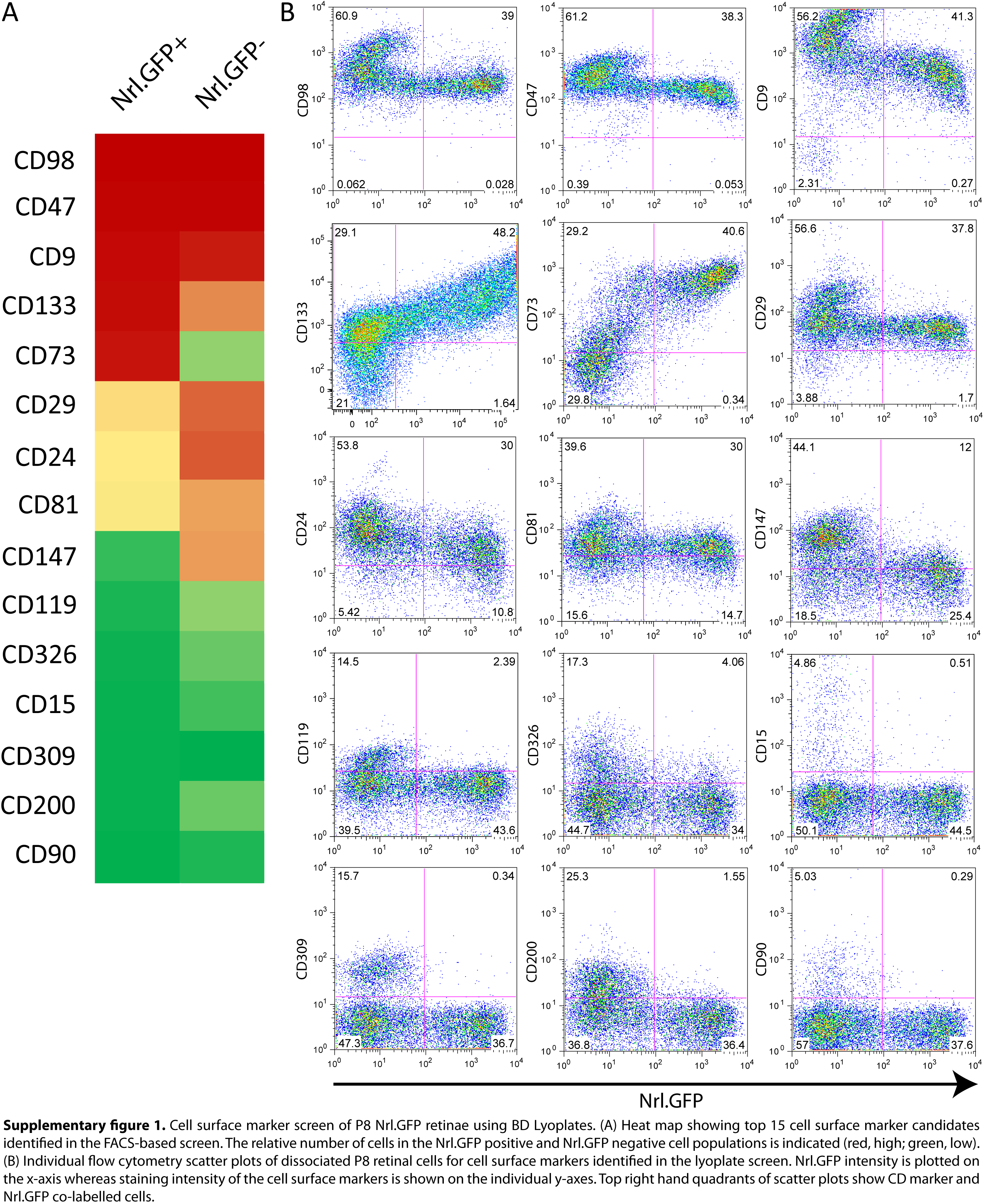

Supplement: Supplementary file 1 — Supplementary Information [file STEM-33-2469-s001.tif]

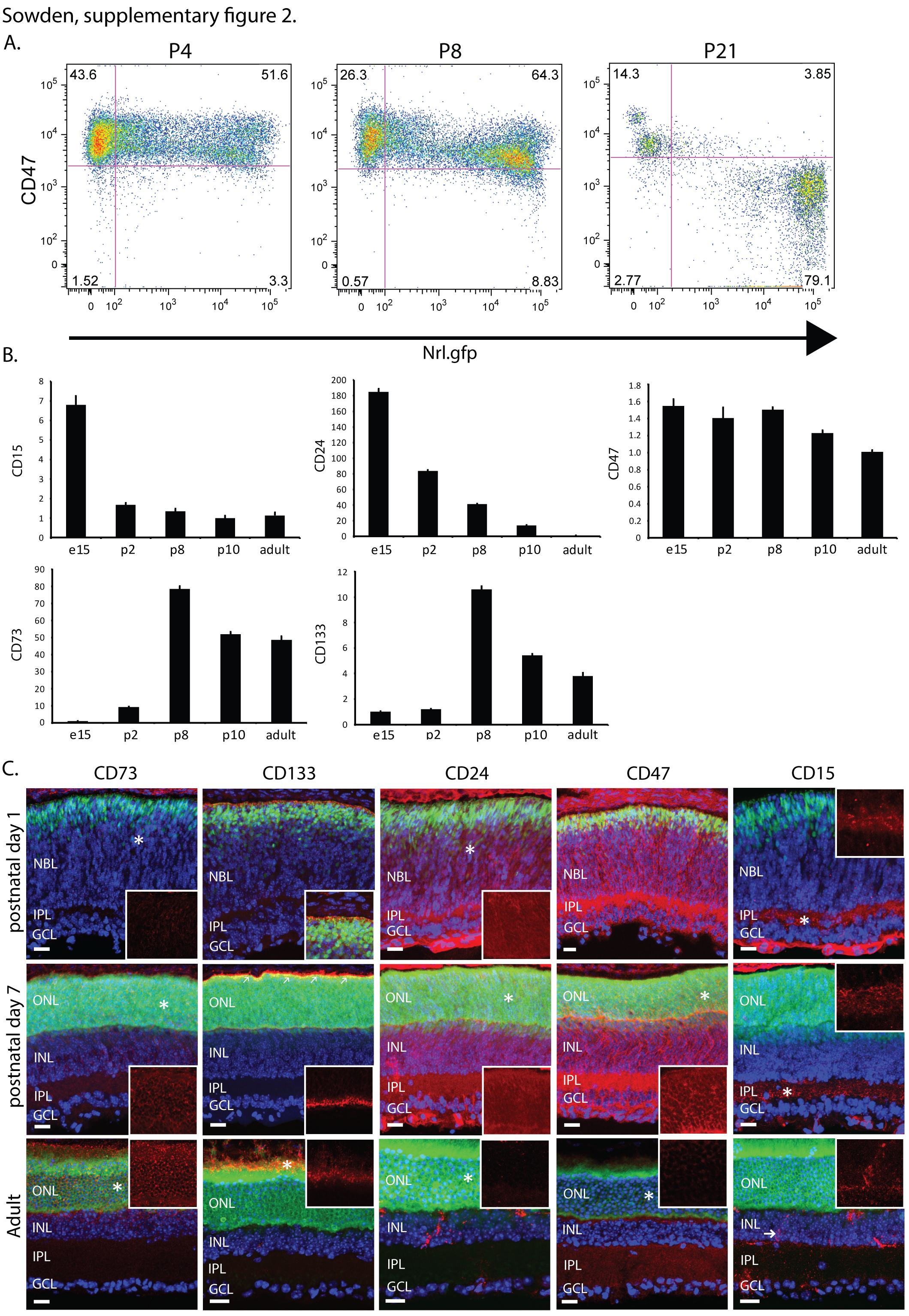

Supplement: Supplementary file 2 — Supplementary Information [file STEM-33-2469-s002.tif]

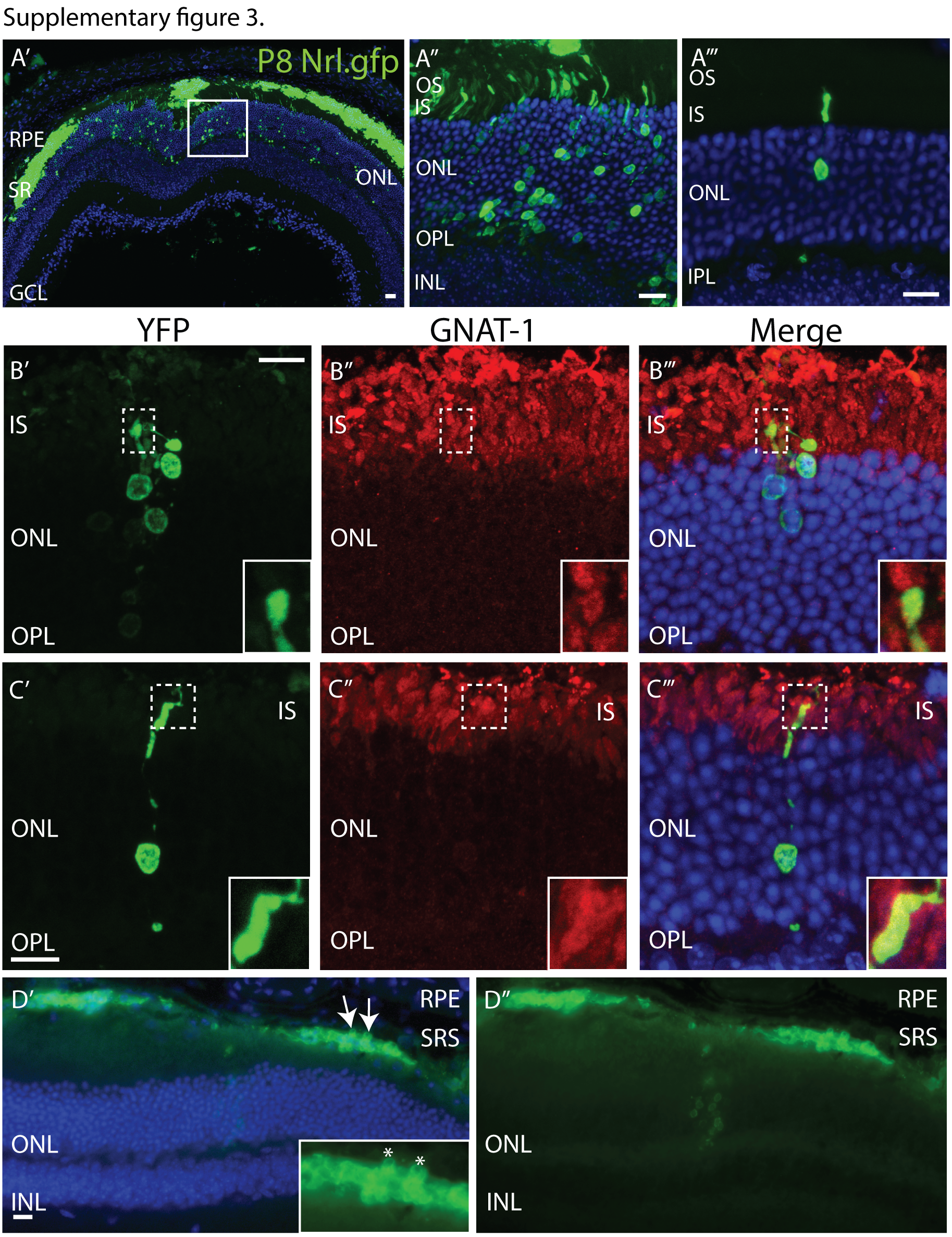

Supplement: Supplementary file 3 — Supplementary Information [file STEM-33-2469-s003.tif]

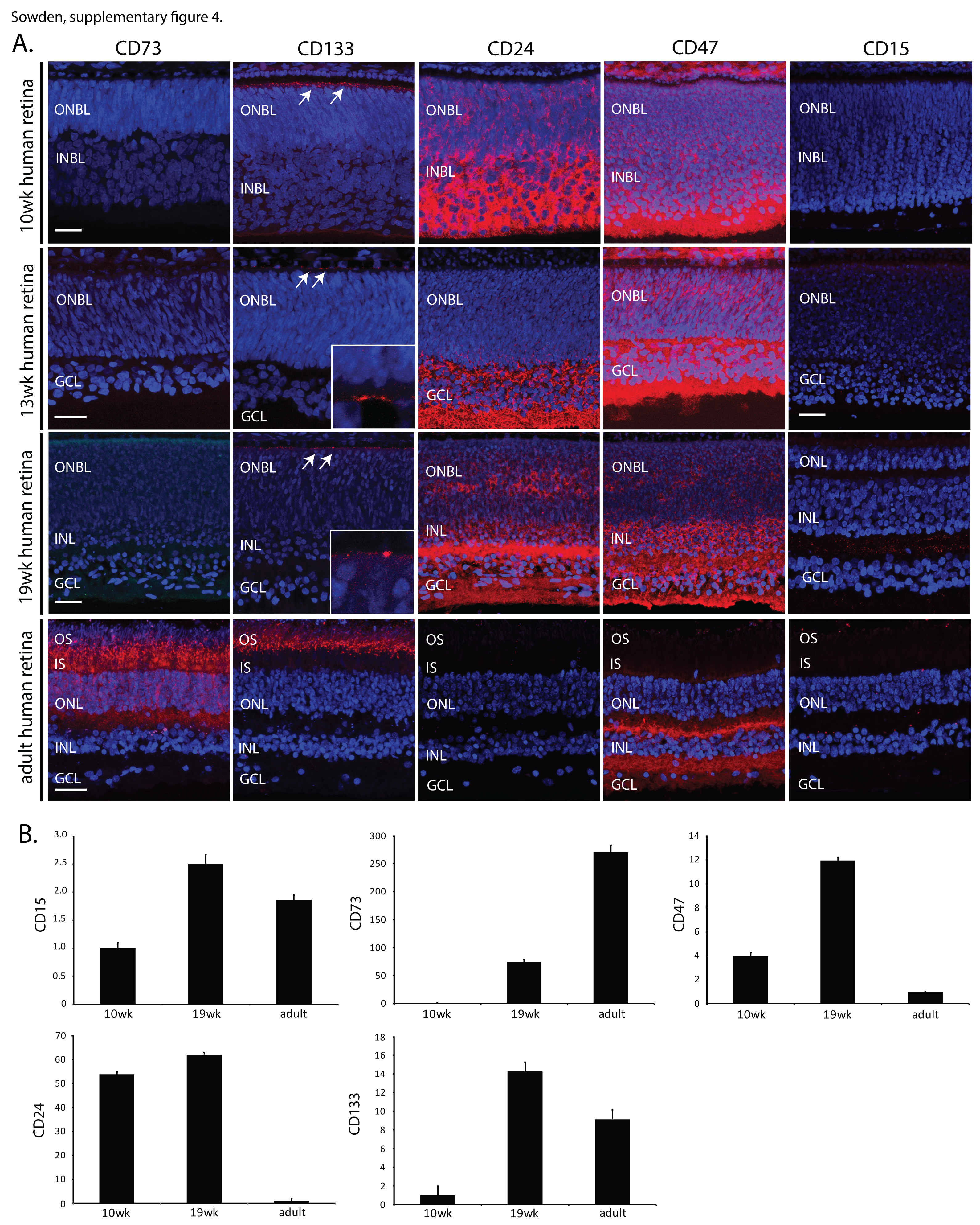

Supplement: Supplementary file 4 — Supplementary Information [file STEM-33-2469-s004.tif]

Sowden, Supplementary Table 1


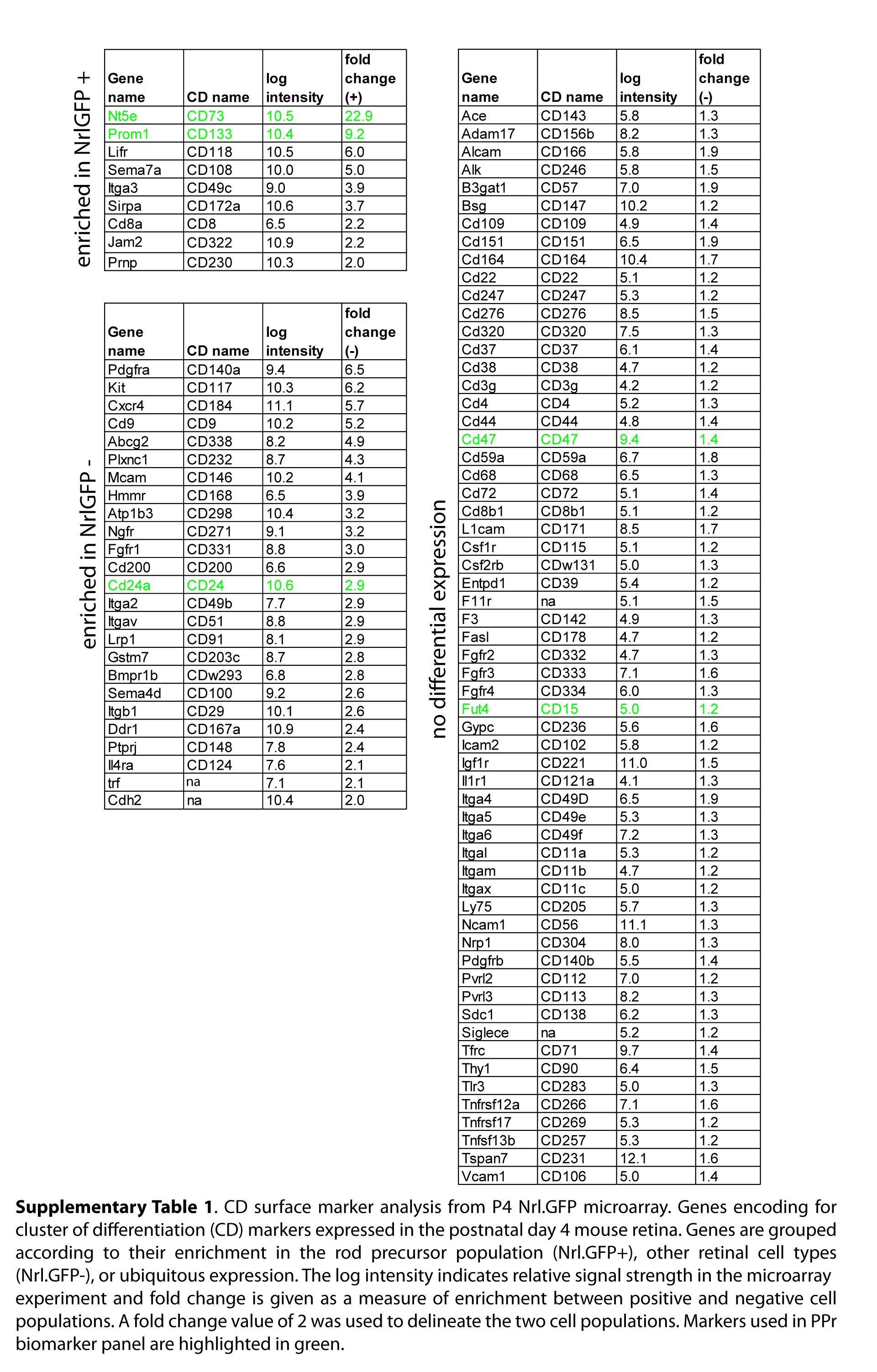

Supplement: Supplementary file 5 — Supplementary Information [file STEM-33-2469-s005.docx]
