# Supplementary material for: Transplantation of Photoreceptor Precursors Isolated via a Cell Surface Biomarker Panel From Embryonic Stem Cell‐Derived Self‐Forming Retina
Source: Stem Cells. 2015 May 27;33(8):2469–82. doi: 10.1002/stem.2051 (PMC4862023; doi:10.1002/stem.2051)
Supplement: Supplementary file 6 — Supplementary Information [file STEM-33-2469-s006.docx]

Supplementary File 1

**Material and Methods**

**Animals**

Experimental mice were kept in University College London animal facilities and all experiments were conducted in agreement with the Animals (Scientific Procedures) Act 1986 and the Association for Research in Vision and Ophthalmology Statement for the Use of Animals in Ophthalmic and Vision Research. *C57Bl/6J,* and *Gnat1^−/−^* (kind gift of J. Lem; [[1](#_ENREF_1)]) recipient mice were between 6 and 10 weeks of age at the time of transplantation.

**Mouse ES cell culture and 3D retinal differentiation**

The mouse EK.CCE ESC line [[2](#_ENREF_2)] (129/SvEv; a kind gift of E. Robertson) or CBA.YFP ESC line (a variant of R1 ESCs; 7AC5/EYFP, from ATCC) were maintained as previously described [[3](#_ENREF_3)]. On day 0 of retinal differentiation, 3 × 10^4^ ESCs were resuspended in one milliliter of differentiation medium (GMEM containing 1.5% KSR, 0.1 mM NEAA, 1 mM pyruvate, 0.1 mM 2-mercaptoethanol) and plated into 96-well low-binding (Corning) plates. Embryoid-body cell aggregates were cultured at 37°C, 5% CO_2_ and growth factor-reduced Matrigel (BD Biosciences) was added on day 1 of retinal culture to a final concentration of 2% (v/v). At day 9, whole EBs were transferred into retinal maturation medium (DMEM/F12 Glutamax containing N2 supplement and Pen/strep), plated in low-binding plates at a density of 6 wEBs/cm^2^ and maintained at 37 °C, 5% CO_2_. The media was changed every 2–3 days and 1 mM taurine (Sigma) and 500 nM retinoic acid (Sigma) were added from day 14 of culture onward.

**Histology and Immunohistochemistry**

Tissue specimens were fixed in 4% (w/v) phosphate-buffered formaldehyde solution at 4°C for 30min, washed three times with phosphate-buffered saline (PBS) and equilibrated in 30 % (w/v) sucrose solution for cryo-protection at room temperature for 1-2 hours. Subsequently, the specimens were transferred into an optimal cutting temperature (OCT)-compound (RA Lamb) filled mould prior to freezing in a dry ice-methylbutane slurry. Tissue sections were prepared on a cryostat (Leica CM1900 UV) to 14-18 μm thickness and collected onto Superfrost^TM^ plus glass slides (VWR). OCT compound was removed by a 15 min incubation in 37 °C PBS. Tissue sections were then blocked with 10 % (v/v) goat serum, 1 % (w/v) bovine serum albumin (BSA) in PBS containing 0.1 % (v/v) Triton X-100 for one hour at room temperature preceding the primary antibody incubation. Triton X-100 was omitted for staining of cell surface molecules. The following primary antibodies were used; Recoverin, Millipore, 1:1000, overnight, 4°C; Cone arrestin, Millipore, 1:20,000, overnight, 4°C; PKCa, Millipore, 1:1000, overnight, 4°C; Prominin 1, Biolegend, 1:350, overnight, 4°C. CD73 Biolegend 1:250, overnight, 4°C or CD24 BD Bioscience 1:250, overnight, 4°C). The primary antibody was omitted for negative controls. Primary antibody staining was followed by several washes with PBS. Tissue sections were then incubated for 1h at room temperature with the corresponding secondary antibody diluted in blocking solution (Goat anti-rabbit AlexaFluor594, Invitrogen, A-11037, 1:300; Donkey anti-sheep Cy3, Jackson, 1:500; Goat anti-mouse AlexaFluor594, Invitrogen, 1:500). Hoechst 33342 (1:3000, Sigma-Aldrich) was applied for 10 min at room temperature to visualize nuclei, followed by three several washes with PBS prior to cover-slipping with the Citifluor AF-1 (Electron Microscopy Science) mounting medium.

**Dissociation of Retinal Cells/ESC retinal cultures and Flow Cytometry**

Neural retinae from wild-type eyes were isolated by micro-dissection and dissociated into a single cell suspension using enzymatic treatment with papain according to the manufacturer’s instructions (Worthington Biochemical, Lorne Laboratories, UK). In the case for mouse ESC cultures, differentiated whole embryoid bodies from day 12 and 27 of differentiation were used for analysis and treated similarly to retinal tissue. Eyes from a variety of developmental stages (E15.5, E17.5) and postnatal day, [P] 4, and [P] 8 as well adult were isolated and dissociated.

Following dissociation cells were resuspended in FACS blocking buffer containing 1% BSA (w/v), phosphate-buffered saline and incubated for 45min on ice. The conjugated antibody or IgG isotype controls were added and cells were incubated in the dark on ice for additional 45min. The conjugated monoclonal antibodies were used for FACS analysis: PE-conjugated CD73 (clone TY/11.8, eBioscience); Phycoerythrin-Cy7 conjugated CD24 (clone M1/69, BD Bioscience); PerCP-eFluor710 conjugated Prominin-1 (CD133, clone 13A4, eBioscience); AlexaFluor 647 conjugated CD47 (Biolegend, miap301); V450 conjugated CD15 (clone MC480, BD Horizon). Antibody specificity for these monoclonal antibodies including Western blot analysis has been previously demonstrated. The same antibody clones were used for FACS and immunohistochemistry analyses. FACS antibodies were used according to manufacturer’s instructions.

After staining the cells were centrifuged at 200g for 5min at 4°C and resuspended in PBS and kept on ice until analysis. FACS analysis was carried out using a BD Bioscience LSR II flowcytometer and FlowJo software (Tree Star, USA). FACS gates were set according to specific isotype controls and at least 20000 events of live cells were analysed. FACS compensation was carried out using BD FACSDiva software using single stained controls for each conjugated antibody. Data presented is from at least 3 biological replicates.

**Immunocytochemistry on dissociated and FAC-sorted ESC-derived cells**

Day 27 ESC retinal cultures were dissociated and sorted via biomarker panel as described above. 50000 cells were plated on poly-lysine/laminin coated coverslips and allowed to adhere for 30min at 37C. Coverslips were then washed once with PBS and adherent cells fixed with 4% PFA/PBS for 10min at room temperature. Following three times washing with PBS, samples were blocking in 10% FBS, 1% BSA/ PBS containing 0.1 % (v/v) Triton X-100 for 1h at room temperature. The blocking solution was replaced by staining solution containing anti-Ki67 antibody in 10% FBS, 1% BSA/ PBS (0.1 % (v/v) Triton X-100). The primary antibody was omitted for negative controls. Finally coverslips with adherent cells were then incubated for 1h at room temperature with the secondary antibody diluted in blocking solution (Invitrogen, Goat anti-rabbit AlexaFluor594) and counter stained 5min with DAPI. The percentage Ki67 positive cells in the experimental groups was established by Cellprofiler analysis software, using confocal tile scans and was verified by manual cell counts; > 100 cells were counted from 3 biological replicates for each condition.

**Microarray and lyoplate and screen for cell surface markers**

Postnatal day 4 Nrl.GFP microarray data were previously published [[4](#_ENREF_4)] and deposited in the National Center for Biotechnology Information’s (NCBI; Bethesda, MD) Gene Expression Omnibus (GEO accession number E-MEXP-3922). Array data were further analysed using Onto-express (<http://vortex.cs.wayne.edu/ontoexpress/>) and DAVID (**D**atabase for **A**nnotation, **V**isualization and **I**ntegrated **D**iscovery; http://david.abcc.ncifcrf.gov/home.jsp), in order to discover genes encoding cell surface CD markers.

Retinal cell suspensions from P8 Nrl.GFP mice were prepared as described above and manufacturers recommendations were followed to conduct the antibody screen using lyoplates (BD). All centrifugation steps were carried out at 300g for 5min at 4°C. After retinal dissociation cells were resuspended in FACS staining buffer (BD) and adjusted to a cell concentration of 10 million cells per 1ml followed by transfer of the cell into round bottom 96-well plates (BD Falcon, Cat. No. 351177). 20 μl of reconstituted primary monoclonal antibody solution was then added to the cells, mixed and incubated on ice for 30 minutes. This was followed by several washing steps with stain buffer (BD Pharmingen) after which the cells were incubated for 30 min with the appropriate biotinylated secondary antibody (rat, 1.25ug/ml; Syrian hamster, 1.25ug/ml; Armenian hamster, 0.6ug/ml; mouse, 1.25ug/ml). Following several washing steps 100 μl of Alexa Fluor® 647 Streptavidin (1:4000, 0.5ug/ml) was added to each well containing cells stained with the biotinylated secondary antibodies and incubated on ice in the dark for 30min. Finally, stained cells were washed several times and analysed on a BD FACSCalibur. At least 30,000 events were collected for the analysis using FACSDiva software and monoclonal antibodies were assessed for their ability to label Nrl.GFP positive rod precursors.

**Cell cycle analysis**

Click-iT EdU analysis was carried out according to manufacturer’s recommendations (Life Technologies). Briefly, whole day 27 embryoid body derived retinal cultures were incubated with 10uM EdU for 2h at 37^0^C. Cells were harvested as described above (Dissociation of Retinal Cells/ ESC retinal cultures and Flow Cytometry) and blocked in 1% BSA in PBS. Cells were pelleted and 100ul fixative (4% PFA) was added followed by a 15min incubation at room temperature. The cells were then washed with 1% BSA in PBS, pelleted and re-suspended in 100ul of 1xClick-iT saponin based permeabilization and wash reagent. After 15min of incubation at room temperature 1x Click-iT reaction cocktail was added to the sample and incubated for 30min at room temperature followed resuspension in wash/permeabilization buffer. FACS analysis was carried out on a BD LSRII using unstained EdU negative cells as a control.

For **Ki67 assay** day 27 ESC retinal cultures were dissociated and sorted via the biomarker panel as described above. 50000 cells were plated on poly-lysine/laminin coated coverslips and allowed to adhere for 30min at 37^o^C. Coverslips were then washed once with PBS and adherent cells fixed with 4% PFA/PBS for 10min at room temperature. Following three times washing with PBS, samples were placed in blocking solution (10% FBS, 1% BSA/ PBS containing 0.1 % (v/v) Triton X-100) for 1h at room temperature. The blocking solution containing anti-Ki67 antibody was then added for 60 minutes at room temperature. The primary antibody was omitted for negative controls. Finally, coverslips with adherent cells were then incubated for 1h at room temperature with the secondary antibody diluted in blocking solution (Invitrogen, Goat anti-rabbit AlexaFluor594) and counter stained for 5min with DAPI. The percentage Ki67 positive cells in the experimental groups was established by Cellprofiler analysis software, using confocal tile scans and was verified by manual cell counts.

**Retinal Cell Transplantations**

Donor cells for subretinal transplantation were derived from either CCE or ATCC-R mouse ESC lines, or from Nrl.GFP postnatal day 8 retinae (Nrl.GFP mice were a kind gift of A. Swaroop; [[5](#_ENREF_5)]), and isolated as described above. For transplantation via PPr biomarkers, cells were incubated in blocking solution (1%BSA/PBS) for 1h and subsequently stained with specific monoclonal antibodies (see above) directed towards the biomarkers according to manufacturer’s recommendations or respective isotype controls. ESC-derived photoreceptor precursors were isolated by FAC-sorting (BD FACS AriaIII) with gating determined for each individual experiment using single stained controls and combined isotype controls. GFP/YFP fluorescence of donor cells was not taken into consideration for cell isolation. Cells in experimental group “unsorted” were processed identically to labelled cells except they were ungated. Post sort cell viability was > 85% based on DAPI staining, and the sorted cells were resuspended at 200,000 live cells/μl in injection buffer (EBSS, DNaseI) after centrifugation at 200g for 10min using a Heraeus Labfuge 400R (Thermos, UK).

Recipient mice (6-8wk, C57Bl/6J or *Gnat1^-/-^*) were anaesthetised with an intraperitoneal injection of 0.2 ml of a mixture of Domitor (1 mg/ml (medetomidine hydrochloride, Pfizer Pharmaceuticals, Kent UK), ketamine (100 mg/ml, Fort Dodge Animal Health, Southampton, UK) and sterile water (ratio 5 : 3 : 42). Topical application of 1% tropicamide was used to dilate pupils of animals and injections were performed using a Zeiss operating microscope. Fundi were visualised using a contact lens system consisting of a coverslip and a drop of coupling medium liquid (Viscotears, Novartis Pharmaceuticals, Frimley, UK). The 34G injection needle loaded with 1 µl of cell suspension (containing 200,000 live FAC-sorted cells) was inserted under direct visualization through the superior equatorial sclera and guided into the sub-retinal space and towards the posterior pole, creating a self-sealing sclerotomy. Injection of the cell suspension in the superior hemisphere resulted in a bullous retinal detachment around the injection site. Anaesthesia was reversed by administration of 0.2ml of Antisedan (atipamezole hydrochloride 0.10 mg/ml, Pfizer, Kent UK). The retinas of recipient mice were harvested 3 weeks post cell transplantation and processed for analysis.

**Counts of integrated photoreceptors**

The number of integrated photoreceptor cells in the ONL of recipient retinae was established by counting serial sections of the eye. CBA.YFP cells with a cell body located within the ONL and displaying at least one of the following structures: inner/outer segment, inner/outer processes, synapse in the OPL, were scored as new integrated photoreceptors. The total number of integrated cells per eye was determined by counting all the integrated CBA.YFP+ cells in alternate serial sections through each eye. All transplanted eyes that contained CBA.YFP cells in the ONL and/or the sub retinal space were included in statistical analyses and all data points are represented in graphs. Mann Whitney tests were used to compare median integration efficiencies between samples.

**Microscopy, Image Acquisition, and Processing**

For epifluorescent analysis retinal sections were viewed on a Zeiss Axioplan 2 and images captured using a Jenoptik C14 digital camera (OpenLab, Improvision). A Zeiss LSM710 (Zen2009, Zeiss) was used for acquisition of confocal micrographs. Images were processed in Zen2009 (Zeiss), Photoshop CS4 (Adobe), Illustrator CS4 (Adobe) and FIJI. Double-labelling analysis was carried out in Adobe Photoshop CS4.

**Transcript analysis by quantitative Real-Time Polymerase Chain Reaction (qRT-PCR)**

Total RNA was extracted from retinal induced embryoid bodies or from FAC-sorted cell populations using the RNeasy Mini Kit (Qiagen, UK). An on-column DNA digest was performed to eliminate all trace amounts of genomic DNA from the samples. Following quantification of total RNA using a NanoDrop ND-1000 spectrophotometer, cDNA was generated by means of M-MLV-reverse transcriptase (Promega, USA). Gene expression levels were established for *Nt5e*, *Cd24a, Cd47, Cd15* and *Prom1* using Applied Biosystems Taqman PCR reagents and probes on a 7500 Real-Time PCR System according to manufacturer’s recommendations. Gene expression data was normalized using Gapdh as a reference. The mean RQ values as well as RQmin and RQmax as measures of variation were calculated using ABI 7500 software 2.0.1.

**Human retinal cultures**

Human fetal retinal tissue was micro-dissected and dissociated using a papain solution according to manufacturer’s recommendation (Worthington Biochemical Corporation, Lorne Laboratories, UK). Cells were seeded on poly-L-lysine (Sigma-Aldrich) and laminin (Sigma-Aldrich, 1mg/ml) coated glass coverslips and cultured in retinal differentiation media containing DMEM-F12 Glutamax (Invitrogen), 1 x N2 and 1 x B27 neural supplements (Invitrogen) and 10% FBS (Invitrogen) as well as penicillin/streptomycin (Invitrogen). Cell culture media was changed every 2-3 days.

**Reference List**

1. Calvert PD, Krasnoperova NV, Lyubarsky AL et al. Phototransduction in transgenic mice after targeted deletion of the rod transducin alpha -subunit. **Proc Natl Acad Sci U S A***.* 2000;97:13913-13918.

2. Evans MJ, Kaufman MH. Establishment in culture of pluripotential cells from mouse embryos. **Nature***.* 1981;292:154-156.

3. Osakada F, Ikeda H, Mandai M et al. Toward the generation of rod and cone photoreceptors from mouse, monkey and human embryonic stem cells. **Nat Biotechnol***.* 2008;26:215-224.

4. Lakowski J, Han YT, Pearson RA et al. Effective transplantation of photoreceptor precursor cells selected via cell surface antigen expression. **Stem Cells***.* 2011;29:1391-1404.

5. Akimoto M, Cheng H, Zhu D et al. Targeting of GFP to newborn rods by Nrl promoter and temporal expression profiling of flow-sorted photoreceptors. **Proc Natl Acad Sci U S A***.* 2006;103:3890-3895.
